# Prof. George Samuel Fehrsen: 26 August 1938 – 22 May 2018 (almost 80 years)

**DOI:** 10.4102/phcfm.v10i1.1883

**Published:** 2018-11-20

**Authors:** Gboyega A. Ogunbanjo

**Affiliations:** 1 Department of Family Medicine and Primary Health Care, Sefako Makgatho Health Sciences University, South Africa

The heights by great men reached and kept were not attained by sudden flight, but they, while their companions slept, were toiling upward in the night. (Henry Wadsworth Longfellow)

**FIGURE 1:**
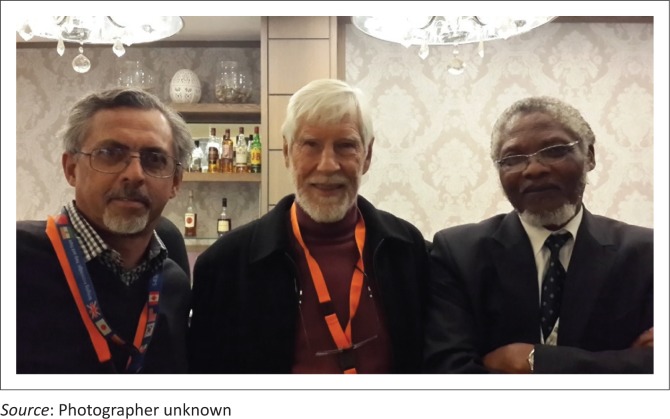
Prof. Samuel Fehrsen (middle), Prof. Graham Bresick (left) and Prof. Khaya Mfenyana (right).

**FIGURE 2:**
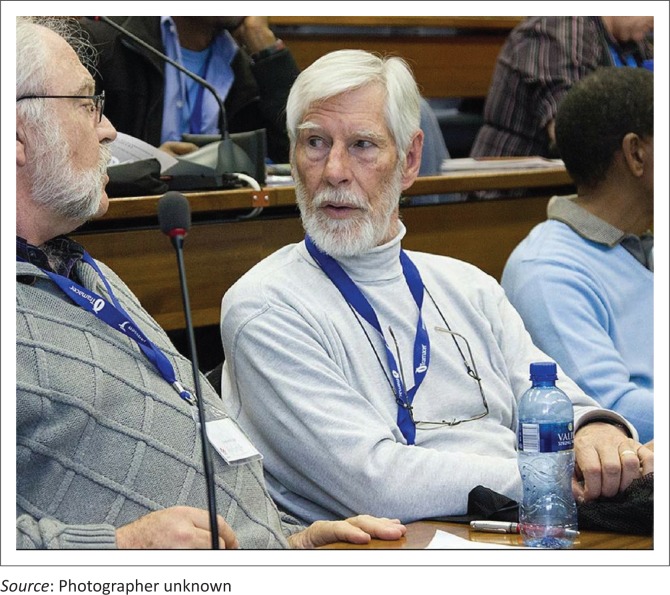
Prof. Samuel Fehrsen.

Prof. Samuel Fehrsen (see Figure 1 and 2) was the eldest of five brothers and one sister and had a happy childhood on a small farm outside Durbanville. He had to travel many kilometres every day to get to good schools for his primary and secondary education. He completed his Bachelor of Medicine and Bachelor of Surgery degrees (MBChB) at the University of Cape Town and qualified as a family physician Member of the Faculty of General Practitioners (South Africa) (MFGP [SA]) with the Colleges of Medicine of South Africa. He worked as a general practitioner for many years in private practice and later in rural district hospitals in the former Transkei including Mount Ayliff Hospital, Eastern Cape, South Africa.

He was the first head of the Department of Family Medicine at the former Medical University of Southern Africa – MEDUNSA (now Sefako Makgatho Health Sciences University) from 1977 to 1996, spanning a period of 20 years. During his tenure at MEDUNSA, Prof. Sam Fehrsen established a family medicine department that trained and produced many family physicians in South Africa, Southern Africa and Central Africa. In 1995, through his visionary leadership, in collaboration with the Evangelical Church of the Congo (ECC), the very first and only Family Medicine postgraduate training programme in the Democratic Republic of the Congo was established. Some of our collaborators from the Democratice Republic of the Congo (DRC) include Prof. Leon Kintaudi and Dr Philippe Lukanu – both based at the Protestant University of the Congo (UPC). The DRC programme has matured to the extent that the department continues to support the local training of family medicine registrars through the establishment of a Memorandum of Understanding with the UPC in 2011.

In addition, he played a pivotal role in the establishment of the first Family Medicine programme in Kenya at the Moi University, Eldoret. Most of the first cohort of family physicians who graduated from that university are now heads of departments (HODs) (Family Medicine) in at least three universities in Kenya. In southern Africa, close to 200 family physicians from Swaziland, Namibia, Lesotho, Zambia and Botswana trained under his leadership through the vocational Family Medicine postgraduate programme at MEDUNSA. A number of family physicians have further emigrated to Canada, Australia and New Zealand. The current HOD of Family Medicine at the University of Namibia (Dr Felicia Christians) is an alumnus of University of Cape Town.

In South Africa, the Family Medicine programme that the late Prof. Sam Fehrsen ran for 20 years produced many prominent family physicians. The current HODs of Family Medicine at Walter Sisulu University (WSU), University of KwaZulu-Natal (UKZN), University of Limpopo (UL), University of the Witwatersrand (Wits) and my humble self (HOD, Sefako Makgatho Health Sciences University [SMU]) are all his mentees and products. He was awarded an honorary degree – Doctor of Medicine (MD) – by the WSU in 2009 for his tremendous contributions to rural health services and training in the former Transkei. What an achievement for someone who was always soft-spoken, affirmative, disciplinarian and a missionary at heart. His command of the isiXhosa language amazed us all the time from his earlier medical professional life at Mt Ayliff Hospital, Eastern Cape. He was a pioneer of the South African Academy of Family Practitioners that later transformed to the South African Academy of Family Physicians, a past president of the Academy, past editor of the *SA Family Practice Journal* as well as a role model, mentor and friend to many. Late Prof. Sam Fehrsen has left footprints on the sands of time that we will never forget. In his quiet way, he succeeded in establishing and spreading the specialty of Family Medicine in South Africa, southern Africa and Africa. May his gentle soul rest in peace!

